# Morphology and phylogeny of the centrohelid heliozoans Raphidocystidae and their ability to consume cyanobacteria

**DOI:** 10.1371/journal.pone.0322585

**Published:** 2025-05-09

**Authors:** Dmitry G. Zagumyonnyi, Yingchun Gong, Da Huo, Denis V. Tikhonenkov

**Affiliations:** 1 Papanin Institute for Biology of Inland Waters, Russian Academy of Sciences, Borok, Yaroslavl Oblast, Russia; 2 Research Center for Aquatic Biodiversity and Eco-Envirnomental Conservation, Institute of Hydrobiology, Chinese Academy of Sciences, Wuhan, China; 3 CAS Key Laboratory of Algal Biology, Institute of Hydrobiology, Chinese Academy of Sciences, Wuhan, China.; VIT University, INDIA

## Abstract

The centrohelid family Raphidocystidae is a large group of heliozoans whose morphology and biology are poorly understood and whose taxonomy and phylogenetic relationships are currently confusing. Here, we report the results of a study of clonal cultures of raphidocystids belonging to the species *Raphidocystis tubifera, R. ambigua, R. marginata* and *R. symmetrica*. The cells were examined using light microscopy as well as scanning and transmission electron microscopy. New data on the morphology of the studied species were obtained and compared with other representatives of Raphidocystidae. For the first time, *R. ambigua* and *R. symmetrica* are reported for Kazakhstan and China, respectively. Cases of taxonomic confusion within Raphidocystidae have been detected and discussed. Molecular phylogenetic analyses based on 18S rRNA sequences clarified the relationships within raphidocystids and confirmed that scales with smooth surfaces are apparently characteristic of the common ancestor of all Raphidocystidae, and scales with a reticular structure were acquired evolutionarily later. Cysts within the Raphidocystidae species were apparently a later evolutionary acquisition. The ability of centrohelid heliozoans to consume cells of toxic and nontoxic cyanobacteria, which cause the most dangerous harmful algal blooms, has been demonstrated for the first time.

## Introduction

Centrohelid heliozoans, also known as centrohelids (Centroplasthelida Febvre-Chevalier and Febvre 1984), are widespread amoeboid heterotrophic axopodial protists with a spherical cell body [1–3]. Centrohelids, together with their closest relatives, haptophyte algae, form the supergroup Haptista [4–6].

Most centrohelids usually have species-specific siliceous surface scales. Although the phylogeny of centrohelids has been partly revised with the introduction of molecular methods based on the 18S rRNA gene sequence [7–11], the ultrastructural features of the scales remain the main criterion for species identification within this group of protists [12–15].

Despite ongoing research on centrohelids, molecular phylogenetic data are still unavailable for many described morphospecies. This, in turn, prevents the qualitative analysis of data from metabarcoding studies, which are increasingly being carried out in freshwater, marine, brackish and hypersaline water bodies and soils [16–24 etc.].

Centrohelid heliozoans are currently represented by two superorders: Panacanthocystida Shɨshkin et Zlatogursky, 2018 and Pterocystida Cavalier-Smith and Heyden, 2007 [7,25,26]. Among Panacanthocystida, 5 families have been identified: 1) Acanthocystidae Claus, 1874 emend. Shɨshkin et Zlatogursky, 2018: cells with an inner layer of oval plate-scales without hollow marginal rim and an outer layer of radially oriented spine-scales with a pronounced basal plate; 2) Marophryidae Cavalier-Smith et Von der Heyden, 2007: cells with numerous thin, pointed organic spicules tapering towards acute apices; 3) Ricksolidae Shɨshkin-Skarð, 2022: cells with an inner layer of oval plate-scales and an outer layer of spine-scales consisting of a bilaterally symmetric basal plate with an axial rib extending over the boundaries of the plate; 4) Yogsothothidae Shɨshkin et Zlatogursky, 2018: cells with two-layered coverings: the inner layer of which is comprised of simple flattened plate scales, and the outer layer is comprised of tangentially oriented scales of various morphologies; 5) Raphidocystidae Zlatogursky, 2018: cells with one type of monolayered plate-scales with hollow marginal rim and sometimes with trumpet-shaped or tubular scales.

The raphidocystid genus *Raphidocystis* Penard, 1904 was established by Eugene Penard [27] and includes heliozoans with a fairly specific morphology and, at the same time, shares characteristics with two other genera known at that time. The first of these is the genus *Acanthocystis* Carter, 1863, with cells having a tight inner layer of overlapping plate-scales and an outer layer of radially oriented spine-scales. The second one is *Raphidiophrys* Archer, 1867, with representatives having similar scattered loose layer of oval, disc-shaped or spindle-shaped scales on the surface. Heliozoans combining both of these traits, that is, having both loose layer of plate scales and spine-scales, were included in the genus *Raphidocystis.* The genus *Raphidocystis* included centrohelids such as *R. tubifera* Penard, 1904, *R. lemani* (Penard, 1891) Penard, 1904, and *R. glutinosa* Penard, 1904, and later, *Raphidocystis glabra* Dürrschmidt, 1985, was added. Most centrohelid heliozoans with tangential plate-scales initially belonged to the genus *Raphidiophrys*.

With the introduction of electron microscopy in the research of heliozoans, it became clear that some species of *Raphidiophrys* have single-layered plate-scales with a rolled edge forming a marginal rim, whereas others have double-layered plate-scales with a marginal rim and series of internal septa [28]. To resolve this ambiguity, Mikrjukov [29] established a new genus, *Polyplacocystis* Mikrjukov, 1996, which included heliozoans with single-layered scales, whereas centrohelids with double-layered scales remained within the genus *Raphidiophrys*. The genera *Raphidiophrys*, *Raphidocystis* and *Polyplacocystis* (as well as *Parasphaerastrum* Mikrjukov, 1996 with a single species) were grouped into the family Raphidiophryidae [29].

Molecular phylogenetic analysis revealed that the genera *Raphidiophrys* and *Raphidocystis* are not closely related. Instead, they form distinct clades of the centrohelid evolutionary tree and belong to Pterocystida and Panacanthocystida, respectively, indicating that they diverged significantly in the past [25,30]. It was also found that the representative of the genus *Raphidocystis* clustered within *Polyplacocystis*, which turned out to be paraphyletic. Therefore, all the representatives of *Polyplacocystis* were transferred to the genus *Raphidocystis*, which has priority [8]. The genus *Raphidocystis* currently includes ten known species, but molecular phylogenetic data are not available for five of them.

The biology of centrohelid heliozoans and raphidocystids in particular has been extremely poorly studied. Centrohelids are known to be predators and are capable of feeding on a wide range of microorganisms [1,2,31]. However, experiments aimed at studying the feeding behaviour of centrohelids are extremely rare [32,33]. Of particular interest is the ability of heliozoans to feed on cyanobacteria. Harmful blooms of cyanobacteria (or blue–green algae) are currently an urgent problem affecting the balance of aquatic ecosystems, the biotic relationships of aquatic organisms and water quality [34]. Cyanobacterial blooms in fresh continental waters can be extremely dangerous and often accompanied by the release of cyanotoxins. For example, *Microcystis* Kützing, 1833 is capable of producing a hepatotoxin called microcystin [35,36]. At the same time, the consumption of photosynthetic organisms by predators is the main mechanism for regulating their numbers and restructuring their populations in natural communities, and heterotrophic protists are the main consumers of cyanobacteria in aquatic ecosystems [37]. Protist grazing has been reported to be effective in reducing both cyanobacterial populations and cyanotoxin concentrations [38,39]. However, nothing is currently known about the potential role of predatory raphidocystids and centrohelid heliozoans in general in the regulation of toxic cyanobacterial blooms.

Here, we report the results of an investigation of the morphology and phylogeny of the 4 species of the genus *Raphidocystis* that have been studied in dynamics in clonal cultures, as well as the results of an experiment on the ability of these species to consume the cyanobacteria *Microcystis aeruginosa* Kützing, 1846 and *Aphanizomenon* sp., which cause the most dangerous harmful algal blooms.

## Materials and methods

### Cultures and samples

Samples containing *Raphidocystis tubifera* (HF-68Z strain) were collected from bottom sediments of the puddle that formed after snow melt in the floodplain of the Ild River, Yaroslavl Oblast, Russia (57°57′26.1″N 38°03′50.1″E), on 07 April 2020. Samples containing *R. ambigua* (HF-58Z strain) were collected from bottom sediments among shoreline aquatic macrophytes of Shchuchye Lake, Aqmola Oblysy, Kazakhstan (52°58′19.60″N 70°14′35.20″E), in August 2018. Samples containing *R. symmetrica* (HF-80Z strain) were collected from bottom sediments among shoreline stands of *Thalia dealbata* Fraser ex Roscoe of Shahu Lake, Wuhan, China (30°34′21.1″N 114°20′37.8″E), on 26 May 2024. Samples containing *R. marginata* (HF-64Z strain) were collected from bottom sediments of Davsha thermal spring, Republic of Buryatia, Russia (54°21′23.0”N 109°29’58.7”E), on 30 August 2019. No permit was required for collection of water samples at these locations.

The samples were transported to the laboratory at 4°C, after which each sample was inoculated into a 60 mm Petri dish. One rice grain was added to stimulate bacterial growth. The samples were incubated at 20–22°C in the dark to prevent the growth of autotrophic organisms and periodically examined with upright and inverted light microscopes. To obtain clonal cultures, single cells of centrohelids were picked out using a pulled glass micropipette with a fine tip and transferred into Petri dishes with autoclaved mineral water (Aqua Minerale, PepsiCo, Inc., Moscow Oblast, Russia). The cell culture of the flagellate *Parabodo caudatus* (Dujardin, 1841) Moreira et al., 2004 (BAS-1 isolate, IBIW RAS), which was grown in the same autoclaved mineral water supplemented with *Aeromonas sobria* bacteria (strain ICISC19, Institute for Cellular and Intracellular Symbiosis Collection, Russian Academy of Science, Russia), was used as prey. Strain HF-58Z was isolated in February 2019 and perished in February 2020. Strain HF-64Z has persisted since March 2020, strain HF-68Z since April 2020, and strain HF-80Z since May 2024.To assess the ability of raphidocystids to feed on cyanobacteria, toxic and nontoxic strains of *Microcystis aeruginosa* (strains 905 and 928, respectively) and *Aphanizomenon* sp. (strain 1399) were used. The strains of cyanobacteria were obtained from the Freshwater Algae Culture Collection, Institute of Hydrobiology, Chinese Academy of Sciences, and were grown on Waris-H medium [40].

### Microscopy

An AxioScope A1 upright light microscope (Carl Zeiss, Jena, Germany) with DIC and phase contrast and water immersion objectives (63×) was used for the observation of living cells, and an inverted microscope Axio Observer 5 (Carl Zeiss, Jena, Germany) with phase contrast (objectives 20×) was used for preparing the cell cultures. Light microscopy images were taken with an MC-20 camera (Lomo-Microsystems, Saint Petersburg, Russia) and an MC-1009/S video camera (AVT Horn; Aalen, Germany).

Preparations for studying skeletal elements were air dried and carried out according to previously described methods [11] and observed in transmission JEM-1011 (Jeol, Tokyo, Japan) and scanning JSM-6510 LV (Jeol, Tokyo, Japan) electron microscopes. The acceleration voltage was 80 kV for TEM and 15–30 kV for SEM. Skeletal elements were measured using ImageJ 1.52a software [41].

### Molecular phylogeny

The centrohelid heliozoans were grown in a clonal culture and collected by centrifugation (1000 × g, room temperature) onto the 0.8 μm membrane of a Vivaclear mini column (Sartorius Stedim Biotech Gmng, VK01P042) (HF-58Z strain) or collected on a polycarbonate membrane filter (pore size of 1 μm) using a 60 ml plastic Luer-Lok™ syringe (BD, New Jersey, Cat. No. REF 309653) and a 25 mm filter holder (Swinnex, Millipore) (HF-68Z strain). Genomic DNA was isolated using the MasterPure™ Complete DNA and RNA Purification Kit (Epicentre, Madison, WI, USA, Cat. No. MC85200). The 18S rRNA gene was amplified using EconoTaq Plus Green 2 Master Mix (Lucigen, Middleton, WI, USA, Cat. No. 30033–1). The eukaryote-specific forward primer Thx25F 5′-CAT ATG CTT GTC TCA AAG ATT AAG CCA-3′ [42] and the centrohelid-specific reverse primer Helio1979R 5′-CAC ACT TAC WAG GAY TTC CTC GTT SAA GAC G-3′ [7] were used for the HF-58Z and HF-68Z strains. To amplify the 18S rRNA gene of the HF-80Z strain, a nested PCR approach was used. Twenty centrohelid cells were picked out using a glass micropipette and added to a PCR mixture containing the forward primer Thx25F and the reverse primer 1801R 5′-TGA TCC TTC TGC AGG TTC ACC T-3′ [43]. The amplification product was used for a second (nested) PCR using the eukaryote-specific forward primer GGF 5′-CTT CGG TCA TAG ATT AAG CCA TGC-3′ and reverse primer GGR 5′-CCT TGT TAC GAC TTC TCC TTC CTC-3′ [44]. The PCR program for amplification was as follows: initial denaturation at 95°C for 3 min; 35 cycles of 95°C for 30 sec, 52°C for 30 sec, and 72°C for 1.5 min; and a final extension at 72°C for 5 min.

The amplified DNA fragments were purified with a QIAquick PCR Purification Kit (Qiagen, Hilden, Germany, Cat. No. 433160764).

The PCR products of the HF-58Z and HF-80Z strains were sequenced via Sanger dideoxy sequencing at Evrogen, Moscow, Russia. The PCR products of the HF-68Z strain were sequenced via Sanger dideoxy sequencing using Applied Biosystems 3500 (Applied Biosystems, USA; Hitachi, Japan) at the Papanin Institute for Biology of Inland Waters, RAS. The PCR products for all the strains were sequenced in one replicate, with the exception of strain HF-80Z, which was sequenced in two replicates with 100% sequence identity, using the primers mentioned above and two universal internal primers, 18SintF (forward): 5′-GGT AAT TCC AGC TCC AAT AGC GTA-3′ and 18SintR (reverse): 5′-GTT TCA GCC TTG CGA CCA TAC T-3′. The resulting sequences were assembled from four overlapping reads using the Geneous R6 ver. 6.0.6 program. The 18S rRNA gene sequences generated in this study were deposited in the NCBI GenBank database under the following accession numbers: PQ530865– PQ530867.

Multiple sequence alignment was constructed using the L-INS-i algorithm in MAFFT ver. 7.475 [45] and trimmed using an automated trimming heuristic followed by a gap threshold filter of 0.7 in TrimAl ver. 1.2 [46]. The aligned sequences were visualized and checked with AliView ver. 1.28 [47]. The resulting alignment consisted of 1586 sites and was used to construct the phylogenetic trees. Phylogenetic trees were reconstructed using Bayesian and maximum likelihood (ML) methods.

The maximum likelihood phylogeny was inferred using IQ-TREE ver. 1.6.12 [48], with 1000 nonparametric bootstrap pseudoreplicates under the best fit model (TN + F + R3 model) determined by the in-built ModelFinder [49].

To infer the 50%-majority rule Bayesian phylogenetic tree, the parallel MPI version of MrBayes ver. 3.2.7a [50] was used with four categories of gamma-distributed among-site rate variation under the GTR + I + GAMMA substitution model. To calculate the posterior probabilities of individual nodes, four independent Metropolis-coupled Markov chains were run for 20 million generations and summarized after the 50% burn-in. The convergence of log-likelihood values and model parameters for chains was verified using a plot and convergence diagnostics provided by the MrBayes sump utility. The average standard deviation of the bipartition frequencies was 0.0046 at the end of the Markov chain Monte Carlo simulations.

### Cyanobacteria feeding experiment

An experiment was conducted to test the ability of raphidocystid heliozoans to consume cyanobacterial cells. Initially, the centrohelids *Raphidocystis tubifera* HF-68Z, *R. marginata* HF-64Z, and *R. symmetrica* HF-80Z were grown on *Parabodo caudatus*. After consuming the bodonid prey and increasing the number of cells, the raphidocystid cells (50–70 cells) were transferred to Petri dishes containing 3 ml of the cyanobacterium *Microcystis aeruginosa* (toxic strain 905, ~ 784123 cells ml^-1^), *Microcystis aeruginosa* (nontoxic strain 928, ~ 596204 cells ml^-1^), and *Aphanizomenon* sp. strain 1399 (2703 colonies/filaments ml^-1^), respectively. The experiment was not carried out for *R. ambigua* HF-58Z due to the loss of the culture.

## Results

The morphological descriptions of the studied strains of *Raphidocystis tubifera, R. ambigua, R. marginata* and *R. symmetrica* are provided below. The present article follows the systematics of eukaryotes by Adl et al. [26]. A system of dots “●” is used to indicate levels of taxonomic ranks from high to low (the more dots there are, the lower the rank of a taxon).

● Diaphoretickes Adl et al., 2012
●● Haptista Cavalier-Smith, 2003
●●● Centroplasthelida Febvre-Chevalier et Febvre, 1984
●●●● Panacanthocystida Shɨshkin et Zlatogursky, 2018
●●●●● Acanthocystida Cavalier-Smith et von der Heyden, 2007 emend. Shɨshkin et Zlatogursky, 2018
●●●●●● Chalarothoracina Hertwig et Lesser, 1874 *sensu* Cavalier-Smith in Yabuki et al., 2012 emend. Shɨshkin et Zlatogursky, 2018
●●●●●●● Family Raphidocystidae Zlatogursky, 2018
●●●●●●●● Genus *Raphidocystis* Penard, 1904 emend. Zlatogursky, 2018

**Fig 1:**
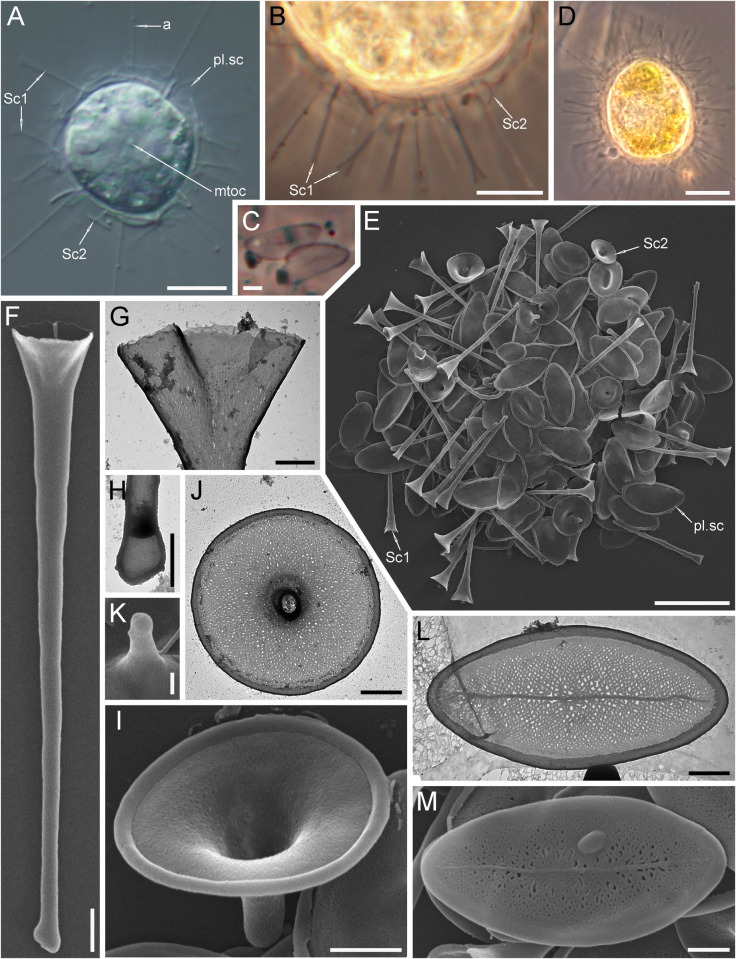
Morphology of *Raphidocystis tubifera.* (A in DIC; B–D in PhC; E, F, K, I, M – SEM; G, H, J, L – TEM). **A**, **B** – living cells; **C** – plate scales; **D** – progenitor cell of the HF-68Z strain; **E** – general view of the dried cell; **F** – trumpet-shaped scale Sc.1; **G** – distal part of scale Sc.1; **H** – proximal part of scale Sc.1; **I**, **J** – funnel-shaped scales Sc.2; **K** – proximal part of scale Sc.2; **L**, **M** – plate-scales. Abbreviations: a – axopodia; sp.sc1 – scale Sc.1; sp.sc2 – scale Sc.2; pl.sc – plate scale; mtoc – microtubule organizing center. Scale bars: A, B, D, E – 10 µm; C – 2 µm; F, I, J, L, M – 1 µm; G, H, K – 0.5 µm.

**Fig 2:**
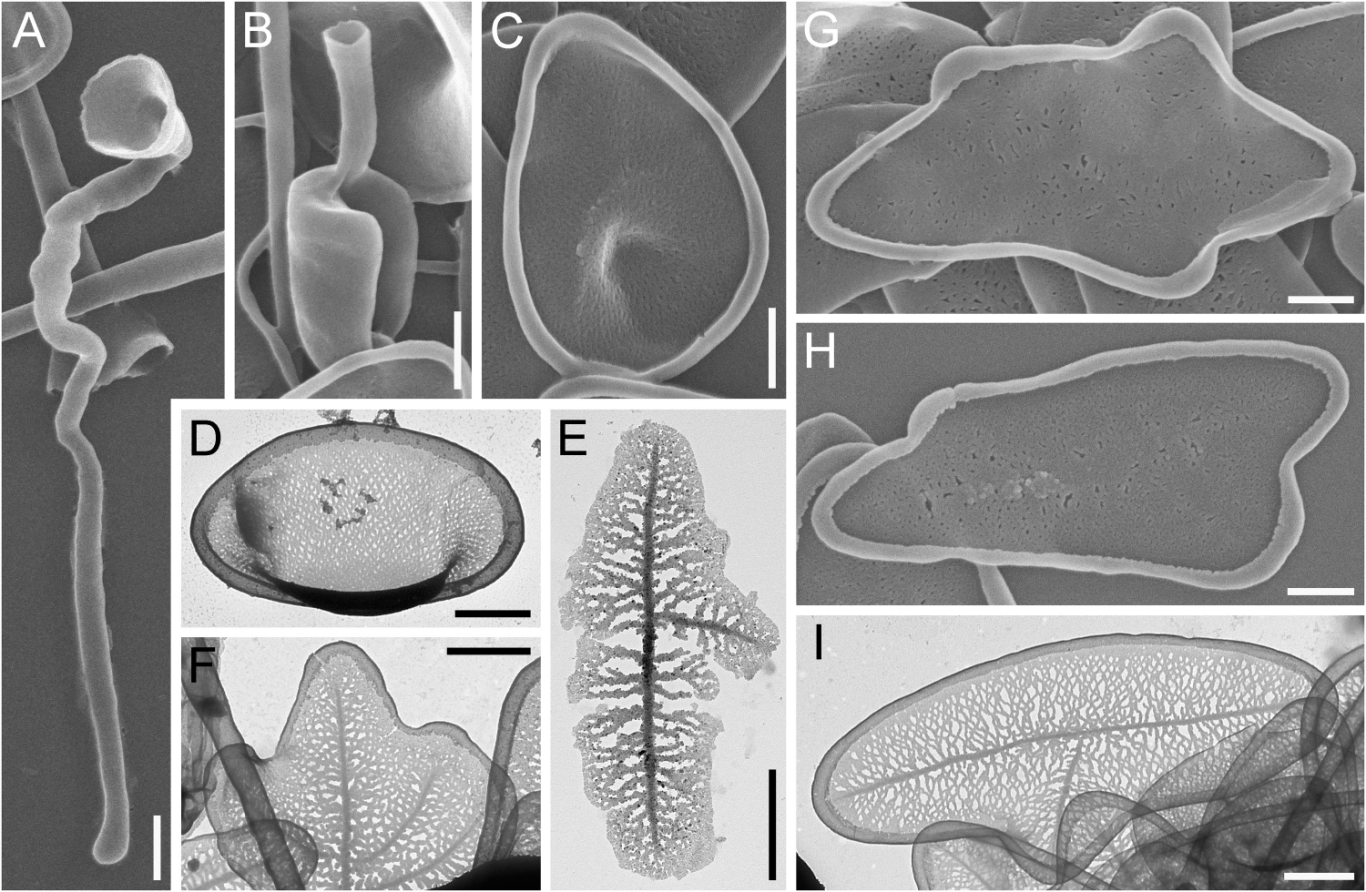
Morphology of *Raphidocystis tubifera.* (A–C, G, H – SEM; D–E, I – TEM) **A**, **B** – Abnormally formed Sc.1; **C**, **D** – transitional type of scale from plate-scales to Sc.2; **E** – underdeveloped teratological plate-scale; **F**–**I** – teratological plate-scales with extra axes. Scale bars: 1 μm.

**Fig 3:**
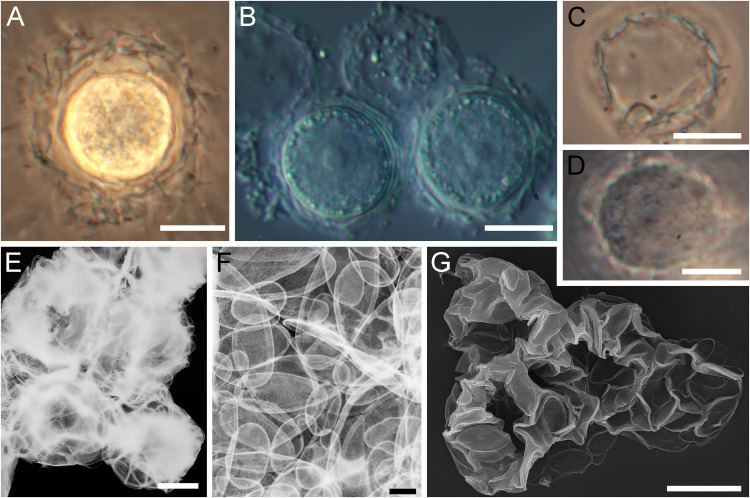
Morphology of *Raphidocystis tubifera.* (A, C, D in PhC; B in DIC; D, E – TEM (inverted); F – SEM) **A** – cyst in early development; **B** – mature cysts; **C–E**, **G** – empty envelopes of dead cysts consisting of plate-scales; **F** – a part of the cyst envelope with small plate-scales. Scale bars: A–D, G – 10 μm; E – 5 μm; F – 1 μm.

*Raphidocystis tubifera* Penard, 1904 (Figs 1‒3, S1 Fig)

**Strain:** HF-68Z

**Description.** The diameter of the live cells is 17.3‒30.4 μm (Fig 1A). The cell surface is covered with silica scales of three types (Fig 1B, C, E). 1) Long trumpet-shaped, radially oriented scales (Sc. 1) are 6.55‒15.60 μm long (Fig 1F‒H). The proximal part of the scale has a small extension 0.24‒0.92 µm wide (Fig 1H), by which it is fastened between the plate-scales. Distally, the scale, which smoothly widens, ends in a funnel with a diameter of 0.97‒4.18 μm. The edges of the distal part are irregular, without a marginal border. 2) The short, funnel-shaped, radially oriented scales (Sc. 2) are 1.69–3.39 μm long (Fig 1I‒K). The distal part has a diameter of 2.61‒5.54 μm and is surrounded by a marginal rim that is 0.15‒0.28 μm wide. 3) Plate-scales 5.26‒9.50 × 2.45‒4.54 μm with axial thickening (sternum) and a broad peripheral rim 0.18‒0.47 μm wide and with a reticulate surface texture (Fig 1L, M). The surfaces of scales Sc. 1 and Sc. 2 also have a reticulate pattern. However, the former appear almost smooth, while the latter have a more pronounced reticular structure.

The cells in culture often exhibit teratological, deformed scales or scales representing transitional variants between the main types of scales (Fig 2). Distorted and abnormally formed Sc. 1 (Fig 2A, B), scales representing a transitional type from plate-scales to Sc. 2 (Fig 2C, D) and plate-scales with extra axes (Fig 2E‒I) were found.

Under unfavourable conditions (lack of food source in old cultures), the cells are capable of forming cysts that are 13.8‒20.9 μm in diameter. (Fig 3A, B). Often, Sc.1 and Sc.2 are absent from the cyst surface, and the cyst wall is represented only by plate-scales tightly cemented together. Even after cell death, the envelope, consisting of plate-scales, retains its shape (Fig 3C‒G). Plate-scales smaller (1.40‒4.90 × 0.82‒2.87 μm) than those on the surface of trophozoites were found on the inner surface of the cyst envelope (Fig 3F).

The progenitor cell of the HF-68Z strain exhibited green inclusions within the cytoplasm, which disappeared in daughter cells after culturing (Fig 1D). The nature of these inclusions remains unclear. Previous studies reported the presence of zoochlorellae in *R. tubifera* cells [27,31,51]. Moreover, Patterson and Dürrschmidt [52] documented that *R. tubifera*, isolated from fresh waters near Bristol, contained functionally intact chloroplasts derived from its algal prey. Therefore, the green inclusions we observed may be ingested algae or zoochlorellae, or they could represent an example of kleptoplasty.Species was previously found in fresh waters of the USA [53–56], Canada [51,57], Peru [58], Argentina [59], presumably in Dominica (according to [60]), Spain (https://www.flickr.com/photos/microagua/27844301899/in/photostream/ accessed 20 June 2024), the United Kingdom [52,57], Switzerland [27], Germany [31], the Netherlands [58,61], Estonia [62], Russia [63–68], presumably in Nigeria (according to [69]), Vietnam [70], Japan (http://protist.i.hosei.ac.jp/PDB/Images/Sarcodina/Heliozoa/Raphidocystis/sp_01.html accessed 20 June 2024), India [71], Australia [72], Tasmania [73], Chile and New Zealand [51], fresh glacial waters of the Greenland Sea [74] and English Channel (salinity ~ 28‰) [75].

**Fig 4:**
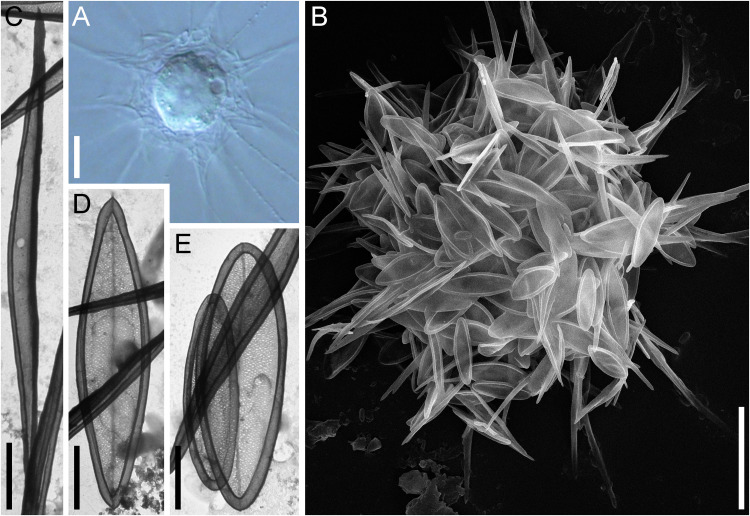
Morphology of *Raphidocystis ambigua* (A in DIC; B– SEM; C–E – TEM) A – general view of the living cell; **B** – general view of the dried cell; **C** – plate-scale of the first type; **D** – plate-scale of the second type; **E** – plate-scales of the third type. Scale bars: A, B – 10 μm; C–E – 2 μm.

*Raphidocystis ambigua* (Penard, 1904) Zlatogursky, 2018 (Fig 4)
Basionym: *Raphidiophrys ambigua* Penard, 1904
Synonym: *Polyplacocystis ambigua* (Penard, 1904) Mikrjukov, 1996

**Strain:** HF-58Z

**Description.** The diameter of the live cells is 15.8–20.4 μm (Fig 4A). The surface of the cell is covered with three types of tangentially located plate-scales (Fig 4B–E). The scales of the first type are narrow and fusiform (9.4–16.0 × 0.6–1.2 μm), with sharp ends and marginal rims 0.17–0.25 µm wide (Fig 4C). The scales of the second type are elongated-oval, 8.4–10.8 × 1.7–2.5 μm with sharp ends and marginal rims 0.24–0.38 μm wide (Fig 4D). The scales of the third type are oval, 4.8–9.5 × 1.7–3.6 μm with rounded poles and marginal rims 0.27–0.39 μm wide (Fig 4E). The inner surface of all scales has a reticular texture. All scales have a well-defined marginal rim and axial thickening (sternum). The plate-scales, especially those of the first type, are arranged along the base of the axopodia. The scales of the second and third types lie tangential to the cell surface. The scales of the first type and some scales of the second type are oriented along the base of the axopodia. Capable of forming cysts according to our observations. The cysts of *R. ambigua* possess a polygonal shape due to the radially oriented plate-scales of the first and second types. Due to the loss of the culture, it was not possible to study the details of the cyst structure.

Previous records were from fresh waters of the USA [56], the Netherlands [28,58,61], France [58], Germany [76,77] and presumably in [78], Ukraine [79–81], Russia; [65,66,82–86] and presumably in [67], Mongolia [87], Canada, Chile, New Zealand and Sri Lanka [51].

This species is reported for the first time in Kazakhstan.

**Fig 5:**
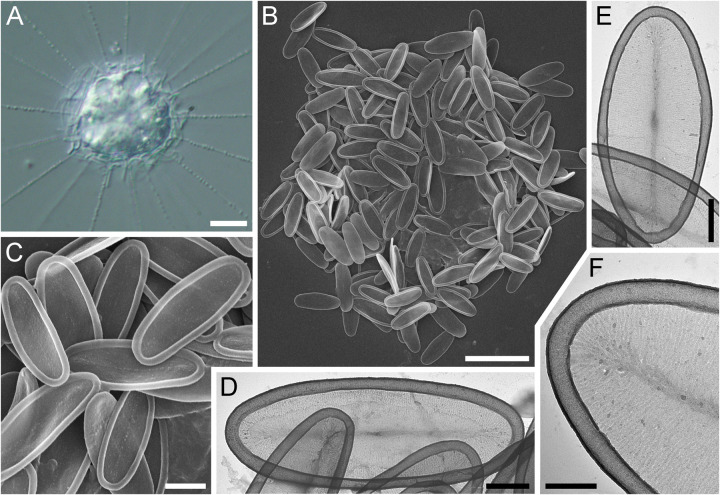
Morphology of *Raphidocystis marginata.* (A in DIC; B, C – SEM; D–F – TEM) **A** – general view of the living cell; **B** – general view of the dried cell; **C–F** – plate-scales. Scale bars: A, B – 10 μm; C – 2 μm; D–F – 1 μm.

*Raphidocystis marginata* (Siemensma, 1981) Zlatogursky, 2018 (Fig 5, S1 Fig)
Basionym: *Raphidiophrys marginata* Siemensma, 1981
Synonym: *Polyplacocystis marginata* Mikrjukov, 1996

**Strain:** HF-64Z

**Description.** The diameter of the live cells is 11.9–25.2 μm (Fig 5A). Skeletal elements are represented by one type of tangentially oriented plate-scales. The scales are not distributed along the axopodia. The plate-scales are elliptical, 4.33–8.32 × 2.20–4.09 μm. The ratio of the length of scales to their width is 1.88–2.86. The surface of the plate-scales appears smooth, with weak axial thickening. At higher magnification, the ribbed texture of the surface is visible, with thin ribs 24–31 nm thick extending from the axial thickening to the marginal rim. The distance between the ribs is 10–15 nm. The hollow marginal rim is 0.21–0.51 μm wide. The formation of cysts was not recorded.

Previous records were from fresh waters of Canada [51], the USA [53,55,56,88], Dominica [60], the Netherlands [28,61], Estonia [62], Croatia [89], Ukraine [33,80], Vietnam [70], Russia [66,67], India [71] and Australia [90], Baltic Sea waters, 6‰ [91], brackish inland water of Russia, 8‰ [65], Moscow State University Marine Aquarium (Russia), 35‰ [92], and the marine environment [93].

**Fig 6:**
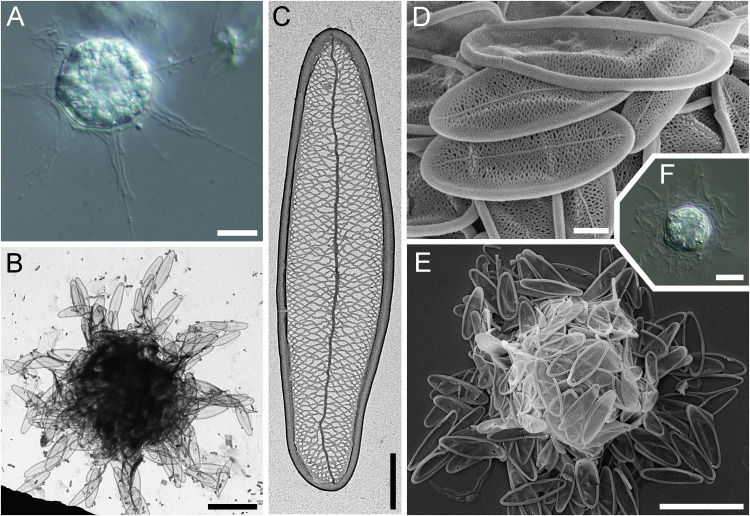
Morphology of *Raphidocystis symmetrica.* (A and F in DIC; B, C – TEM; D–E – SEM) **A** – general view of the living cell; **B** – general view of the dried cell; **C**, **D**– plate-scales; **E**, **F** – cysts. Scale bars: A, B, E, F – 10 μm; C, D – 1 μm.

*Raphidocystis symmetrica* (Penard, 1904) Zlatogursky, 2018 (Fig 6, S1 Fig)
Basionym: *Raphidiophrys symmetrica* Penard, 1904
Synonym: *Polyplacocystis symmetrica* (Penard, 1904) Mikrjukov, 1996

**Strain:** HF-80Z

**Description.** The diameter of the live cells is 11.9–27.5 μm (starving cells 11.9–17.2 μm; well-fed cells 15.3–27.5 μm). The surface of the cell is covered with elongate-elliptical scales. The scales are evenly distributed both around the cell body and lining the bases of the axopodia (Fig 6A, B), similar to *R. ambigua*. The scales are 5.56–10.45 ×1.41–3.25 μm in size (Fig 6C, D). The scale length-to-width ratio is 2.1–4.8. The marginal rim is 0.19–0.38 μm width. The inner surface of the scales exhibits a distinct reticular texture and well-defined axial thickening (sternum).

The species is capable of forming cysts 8.7–10.9 μm in diameter (Fig 6F, E) without forming additional cyst scales that differ in morphology from those of the trophic phase.

Species was previously found in fresh waters of Switzerland [27], Germany [31], Canada [94], the Netherlands [28,61], the United Kingdom [95], Ukraine [81], Russia [12,83,86,96] and presumably in [66], Vietnam [70,97], Australia [73], presumably in Japan (according to [98]) and fresh glacial waters of the Greenland Sea [74].

This species is reported for the first time in China.

### Phylogenetic position

**Fig 7:**
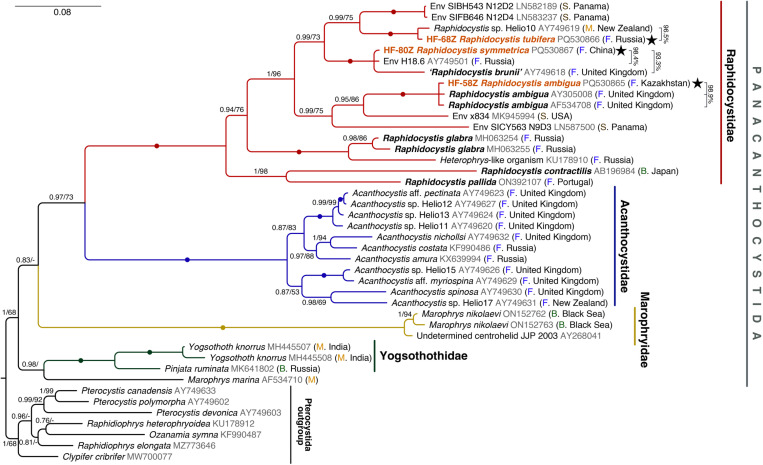
Bayesian phylogenetic tree generated from 18S rRNA gene sequences of 42 centrohelids (seven of which represent the outgroup). Bayesian posterior probabilities (BPP) and Maximum Likelihood (ML) bootstrap values are indicated at branches (BPP > 0.50 and ML bootstrap > 50% are shown; dt – different topology). Filled circles of different colours indicate values of BPP = 1.00 and ML bootstrap = 100%. The sequences generated in this study are highlighted in bold, colored in red and marked with black stars symbols. The percentage of similarity of the sequences is indicated to the right of the names of the sequences. Abbreviations: B, brackish environment: F, freshwater environment; M, marine environment; S, soil environment.

The three molecularly characterized centrohelids in this study belong to the family Raphidocystidae: Panacanthocystida (Fig 7). *Raphidocystis tubifera* HF-68Z is grouped with full support with the unidentified marine centrohelid Helio10 (AY749619) from Whale Bay, Raglan, New Zealand, with which they are 96.5% similar. Two environmental sequences from the rainforest of Panama are grouped together in a sister clade.

In the sister group to the clade of the four above-mentioned*, R. symmetrica* HF-80Z clustered with full support with the freshwater sequences H18.6 (AY749501) and “*R. brunii*” (AY749618). A discussion of the cospecificity of these strains is given below. BLASTn analysis revealed 98.4% similarity between HF-80Z and H18.6 and 93.3% similarity with “*R. brunii*”.

The HF-58Z strain with complete support was grouped within a clade with other representatives of *Raphidocystis ambigua* from the United Kingdom (AY305008 and AF534708). The similarity between these sequences is 98.9%. The environmental sequences from the soils of the USA and Panama were placed more basally.

### Feeding of raphidocystids on the toxic and nontoxic freshwater cyanobacteria

**Fig 8:**
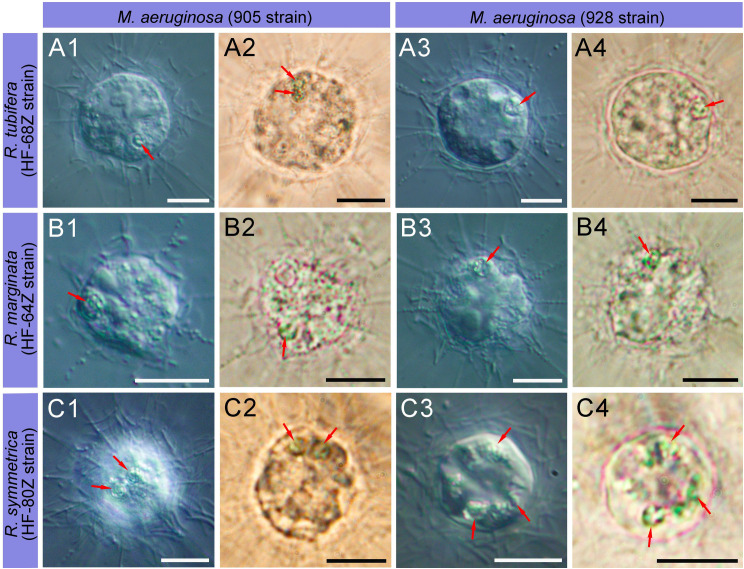
Feeding of raphidocystid heliozoans by cyanobacteria. (A1, B1, C1, A3, B3, C3 in DIC; A2, B2, C2, A4, B4, C4 in bright field). **A1–A4** – *R. tubifera* HF-68Z with ingested cells of *M. aeruginosa*; **B1–B4** – *R. marginata* HF-64Z with ingested cells of *M. aeruginosa*; **C1– C4** – *R. symmetrica* HF-80Z with ingested cells of *M. aeruginosa*. Red arrows show ingested cyanobacterial cells. Scale bars: 10 μm.

All tested strains of raphidocystids showed significant uptake of cells of both the toxic and nontoxic strains of *Microcystis aeruginosa* (Fig 8). At the same time, the density of the cyanobacterial cell suspension did not noticeably decrease. The death of heliozoans after their transfer to cyanobacteria was not recorded. Cell divisions of heliozoans were not observed in cyanobacterial cultures. On the 10th day of observation, in all the cases, active cells of raphidocystids with elongated axopodia were recorded. When the feeding intensities of raphidocystids on bodonid and cyanobacterial cultures are compared, more active feeding is observed on the flagellates *Parabodo caudatus*. In this case, raphidocystid cells, which simultaneously consume up to ten bodonid cells, were observed. During feeding on cyanobacteria, despite their high density, a maximum of 3–4 cyanobacterial cells were simultaneously consumed by heliozoan cells. At the same time, interactions between the kinetocysts of heliozoan axopodia and the sheaths of cyanobacteria have never been observed. It is likely that the absorption of cyanobacteria can occur through close contact of their cells with the surface of the centrohelid cell body without anchoring the cyanobacterial cells by the kinetocysts of axopodia, probably due to the presence of a complex cell wall and extracellular covering in *Microcystis*, which includes a peptidoglycan layer and polysaccharide fibrils [99]. In an experiment using *Aphanizomenon* sp., feeding on cyanobacteria was not recorded for any species of raphidocystid heliozoans. At the same time, the trichomes of *Aphanizomenon* sp. were used by raphidocystids as a substrate on which they anchored.

## Discussion

### Morphology and phylogeny of raphidocystidae

A comparison of the literature data revealed some differences between strain HF-68Z and previously studied *R. tubifera* strains. Thus, previous studies reported larger Sc.1 scales in other samples, ranging from 15–25 μm [57], 20.0–37.5 μm [65], 9.70–46.53 μm [70], and up to 39 μm in the meromictic lake Trekhtsvetnoe (White Sea) (Y. Mindolina, personal comm.). Our study also revealed larger plate scales, with lengths reaching up to 9.50 μm and widths up to 4.54 μm, than those reported in previous studies by Croome [72], Mikrjukov [62], and Rees et al. [57]. These studies reported maximum lengths of 7.5 μm, 4.8 μm, and 6.1 μm, and maximum widths of 2.3 μm, 2.0 μm, and 3.1 μm, respectively.

The scale of the transitional shape compared with Sc.2 and plate-scales (Fig 2D) is marked in Fig 5 in [51]. Notably, in samples studied directly from the environment [57,70,72 etc.], the amount of Sc.1 is usually greater than that in samples cultivated in the laboratory for 4 years (HF-68Z strain).

Sequencing of the studied clones provided the first insights into the phylogenetic placement of *R. tubifera* within the centrohelids. The assumption of Zlatogursky et al. [8] about the phylogenetic position of *R. tubifera* close to *R. ambigua* and ‘*R. brunii*’, which possess plate scales with a reticular texture, was confirmed.

It is worth noting that the sequences closest to *R. tubifera* were isolated from significantly different biotopes. Strain HF-68Z was isolated from a freshwater biotope, sequence Helio10 from seawater, and the sequences LN582189 and LN583237 from the tropical forest of Panama. The vast majority of findings of *R. tubifera* were made in freshwater biotopes. However, Tong [75] noted two scales of *R. tubifera* from Southampton water (salinity ~ 28‰) north of the Solent. Apparently, they may belong to the freshwater heliozoans brought by the River Test current. Nonetheless, the presence of the close sequence of ‘Helio10’ from the sea waters of Whale Bay, New Zealand, allows us to assume the existence of a closely related species to *R. tubifera*, adapted to living in sea water.

The type species of the genus *Raphidocystis*, *R. lemani* (Penard, 1891) Penard, 1904, is quite morphologically similar to *R. tubifera* and is not often noted by researchers. The morphology of the covering scales, as well as their variability, is poorly studied in *R. lemani*. This species must be isolated in clonal culture to obtain molecular phylogenetic and detailed morphometric data. On the basis of the available morphological data, this species is expected to form a lineage close *to R. tubifera* in future phylogenetic reconstructions.

The revealed morphological characteristics of clone HF-58Z generally coincide with those described previously for *R. ambigua* [1,2 etc]. This species is reported for the first time for the water bodies of Kazakhstan. The similarity of the sequence of our strain with the available *R. ambigua* sequences is also very high (98.9%). The sequences AY305008 and AF534708 belong to the same strain from River Cam, Cambridgeshire, UK. It was deposited in the CCAP culture collection (1568/1) and subsequently sequenced independently twice [3,100]. The obtained sequences of the strains were 99% similar to each other, with 11 nucleotide substitutions out of 2209 nucleotides. NCBI also currently includes the sequence LC682598 from Wake city, Okayama, Japan, which is annotated as *Raphidocystis ambigua*. The length of the LC682598 sequence is significantly shorter than that of sequences from the UK and Kazakhstan (1240 bp). When the tree is reconstructed, this sequence forms a long branch, grouping within a clade with other *Raphidocystis ambigua* sequences.

A number of problems have been accumulated regarding *Raphidocystis marginata* and morphologically similar centrohelids. This species was first described in 1981 by F. Siemensma on the bases of light microscopy (as *Raphidiophrys marginata*). Soon after, Nicholls and Dürrschmidt [51] reported specimens with slightly larger scales, but well consistent with the shape and structure of the scales of *R. marginata* and studied them using electron microscopy. Siemensma and Roijackers [28] subsequently confirmed the morphology refined by Nicholls and Dürrschmidt by obtaining electron microscopic data for this species from the Netherlands. Soon, information appeared about brackish-water (Baltic Sea, 6‰, [91]) and marine (35‰, [92]) forms, which were also identified as *R. marginata*.

In 1995, Kinoshita and colleagues described a new species, *Raphidiophrys contractilis*, similar to *R. marginata*, from a brackish water pond (3.5‰), justifying the novelty by the smaller scale and cell sizes and by the absence of broadly rounded poles of plate-scales, although in the provided Figs 4–5, poles are broadly rounded [101]. Mikrjukov in his monograph [2], carried out a revision, transferring *R. marginata* and *R. contractilis* to the previously described genus *Polyplacocystis* as synonyms of *Polyplacocystis coerulea* (Penard, 1904) Mikrjukov et Croome, 1998. The latter was described from the fresh waters of Switzerland as a small (13–14 µm) heliozoan [27], but there were no data on the structure of its scales. It was noted that the cell covers consist of small ‘sequins’ or very thin elongated rods that are straight or slightly curved [27]. Due to the fact that neither the shape of the scales nor their sizes nor any of their elements (e.g., margins, ribs, surface texture, etc.) were described and the type was not preserved, the specimen found by Penard is currently impossible to adequately compare with any known species. After the phylogenetic position of the genus *Raphidocystis* was clarified [8], both *Raphidiophrys marginata* and *Raphidiophrys contractilis* were transferred to *Raphidocystis*.

Analysis of the currently available morphological data does not allow us to clearly draw a boundary between the abovementioned raphidocystid species, since the quality of the data varies greatly among different authors. A number of works present only single scales without describing the morphology of the covering elements [53,55,56,60,66,67,71,88,89,93]. Other works contain only images of living cells [33,90], whereas others present the morphometry of single specimens [70].

The morphometric characteristics of the studied *R. marginata* strain are similar to those previously reported [28,51]. Sharp differences in scale morphology were noted for a strain from a marine aquarium studied by Mikrjukov [92]. This strain is characterized by a more elongated and narrow shape, greater length (8.0–9.0 µm) and smaller width (1.7–2.0) and, accordingly, a greater length-to-width ratio (3.4–5.4 [measurements according to Fig 4 in [92], n = 11] versus 1.9–2.9 in our study). In our opinion, it is another species of *Raphidocystis* closely related to *R. marginata*. Another strain isolated from the Black Sea and considered *R. marginata* by Mikrjukov (Fig 11 in [29]), in our opinion, also belongs to other species owing to an even greater length-to-width ratio (8.7, measurements according to Fig 11 in [29]; n = 1]). In addition to differences in the morphology of the covering elements, the abovementioned species were isolated from marine waters (35‰ and 18‰, respectively), whereas the HF-64Z clone is freshwater.

**Fig 9:**
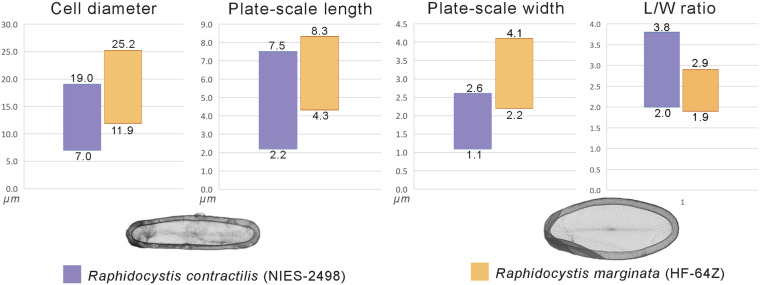
Comparison of the morphometric parameters of *Raphidocystis contractilis* strain Nies-2498 (Wan et al. 2023) and *R. marginata* strain HF-64Z.

Although *R. marginata* strain HF-64Z and *R. contractilis* strain NIES-2498 [101,102] have similar plate scales, the size limits for both scale length and width and cell diameter are lower in *R. contractilis* (Fig 9). The length-to-width ratio in the original description of *R. contractilis* is almost the same as that of the HF-64Z strain (2.0–3.0), but in the subsequent work [102], the microphotographs demonstrate a significantly greater length-to-width ratio (up to 3.8). Therefore, considering the differences in the size and shape of the scales and the differences in the mineralization of the biotopes from which the strains were isolated, we are not inclined to equate these species.

The morphology of *‘Polyplacocystis’ coerulea* revealed by Plotnikov and Ermolenko [65] is closer to that of strain HF-64Z, but this specimen was found in a lake with higher salinity (8‰) than our strain and *R. contractilis* sensu Kinoshita et al. (1995). Therefore, to gain a deeper understanding of the taxonomy and phylogeny of raphidocystids, along with molecular genetic data, salinity experiments are needed to test whether freshwater strains are able to successfully survive and reproduce in brackish-water environments and, vice versa, brackish-water strains in freshwater conditions.

We observed thin ribs on the scales of our *R. marginata* strain, a feature not previously described in the literature. Although not previously reported, these structures are clearly discernible in the provided images (Fig 4D, [89]; Fig 28, [28]).

The morphometric parameters of the studied *R. symmetrica* strain also generally correspond to those previously described for other strains. However, the cells we studied possess slightly larger lengths, up to 10.45 μm versus 9.6 μm in [86], and 8.4–8.7 μm in other studies [12,70,83,96,97], which can probably be explained by the larger sampling size of the current study.

**Fig 10:**
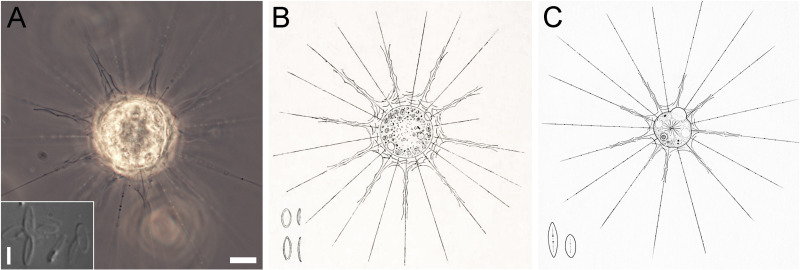
General view of living cells of *Raphidocystis symmetrica.* **A** – *R. symmetrica* HF-80Z strain (PhC), the inset shows plate-scales (DIC); **B** – drawing of *R. symmetrica* (182 p., as *Raphidiophrys symmetrica*) by Penard [27]; **C** – drawing of *R. symmetrica* (77 p., Plaat 19, as *Raphidiophrys symmetrica*) by Siemensma [61]. Scale bar: A –10 μm, inset – 5 μm.

The average size of the well-fed *R. symmetrica* HF-80Z cells was 20.7 µm, matching the average size in the original description (20–25 µm), which was based on light microscopy data only [27]. The general appearance of the cells we studied and the arrangement of their scales at the bases of the axopodia also correspond to those shown by other authors (Fig 10). Penard [27] described two types of scales: long scales located at the base of the axopodia and short scales covering the cell body. In the strain we studied, the distributions of scales of different lengths were the same, but no division of scales into two clear size classes was revealed.

The strain of *R. symmetrica* we studied forms a trichotomy with the environmental sequence AY749501 and the strain identified as ‘*R. brunii’* on the phylogenetic tree. The latter species was identified by Cavalier-Smith and Chao [103] on the basis of cell size and the number of axopodia. The 18S rRNA sequence of this strain was obtained previously [7]. The available microphotographs (Fig 8 in [103]; Fig 4 in [7]) show that the cell covers consist of one type of scales with a reticular surface texture, characteristic of *R. ambigua*, *R. tubifera* and R*. symmetrica*. Unfortunately, the original description of *R. brunii* [104] does not contain enough information about the scale structure, so it is difficult to accept the conclusion of Cavalier-Smith and Chao [103] about the cospecificity of the clone they studied and *R. brunii*. Cavalier-Smith and Chao [103] do not provide scale sizes, preventing comparison with the strain we studied. *R. brunii* is characterized by a cell size of 12–13 µm according to the original description [104]. In the existing figure (Fig 8 in [103]), the cell diameter is approximately 12 μm, which also corresponds to the lower limit of the cell diameter of the strain we studied. The similarity of the 18S rRNA gene sequences of the clone HF-80Z and the so-called “*R. brunii*” is only 93.3%, suggesting that they are two different species since the difference threshold for many described centrohelid species is typically on the order of 3%. It should be noted that Mikrjukov [105] and Prokina et al. [106] considered marine centrohelids with very narrow scales as *R. brunii* (*R. brunii* sensu Mikrjukov 1999 and *R. brunii* sensu Prokina et al. 2021).

Importantly, species with a reticular scale surface structure (*R. tubifera*, *R. ambigua*, *R. symmetrica* and *R. brunii* sensu Cavalier-Smith and Chao 2012) form a distinct clade on the Raphidocystidae tree. In contrast, species with smooth surfaces of scales (*R. pallida, R. contractilis,* and *R. glabra*) are placed closer to the base of the raphidocystid tree. Interestingly, when *R. ambigua* forms cysts, it can also form plate scales without a reticular texture [107]. Therefore, scales with smooth surfaces are apparently characteristic of the common ancestor of all Raphidocystidae, which confirms the view of Drachko et al. [108], and scales with a reticular structure were acquired evolutionarily later.

Cysts within Raphidocystidae species were apparently a later evolutionary acquisition too. Only *R. tubifera*, *R. ambigua* and *R. symmetrica* possess the ability to encyst. While in *R. pallida*, *R. contractilis* and *R. glabra,* the presence of cysts has not been revealed. Cyst formation was also not observed in *R. marginata* in this study. The ability to form cysts in raphidocystids was likely acquired in connection with the transition to life in conditions with variable moisture levels. For example, it is known that *R. tubifera*, *R. ambigua* and *R. symmetrica* can survive in the conditions of temporary waterbodies (HF-68Z in this study), dry mosses (*R. ambigua* [58]), and soils (*R. symmetrica,* unpublished data).

Despite the fact that representatives of Raphidocystidae (e.g., *Raphidocystis glabra* and *R. contractilis*) are characterized by the replacement of siliceous skeletal elements with organic spicules during their life cycle [8,102], this phenomenon was not recorded in our study.

### Feeding on cyanobacteria

The results of the feeding experiment revealed the ability of centrohelids of the genus *Raphidocystis* to use cyanobacteria as food. That is, the abundance of cyanobacteria may potentially be subject to ‘top-down control’ by raphidocystids in aquatic communities. Similarly, feeding on cyanobacteria has been shown for the naked amoebae *Mayorella* [109], *Hartmannella*, *Vexillifera*, *Vannella*, *Korotnovella*, *Copromyxa* [110], *Acanthamoeba castellanii* [111], *Naegleria* [112], heterolobose *Vrihiamoeba* [113], vampirelids [114] and some others. It has been found that mixotrophic representatives of the chrysophytes *Ochromonas* and *Poterioochromonas malhamensis* (Pringsheim 1952) Andersen, Graf, Malakhov et Yoon, 2017 can quickly consume toxic *Microcystis* cells and effectively destroy both intracellular and extracellular microcystins [38,115–120]. Interestingly, heterotrophic protists can be highly resistant to cyanotoxins and exhibit feeding strategies that differ significantly from those of metazoans [39]. Thus, heterotrophic protists have great potential for controlling harmful cyanobacterial blooms and restoring the phytoplankton composition in eutrophic waters. Regarding the results of this study, it should be emphasized that raphidocystids do not consume the filamentous colonies of *Aphanizomenon* sp., and although they are able to consume *Microcystis* cells, their number is not significantly reduced. Further research is needed to examine the conditions that increase cyanobacterial feeding in *Raphidocystis*. This could be useful for the development of biological methods and technologies for the control of toxic cyanobacterial blooms, which are potentially less harmful to the environment and may represent a cost-effective alternative to physical and chemical methods.

## Supporting information

S1 Fig General view of living cells of raphidocystids. *Raphidocystis tubifera* (A–B), *R. marginata* (C–D), and *R. symmetrica* (E–F). A, B, D, F – PhC; C, E – DIC. Scale bars: 10 μm.
(TIF)

S1 Data Morphometric parameters of studied strains of the genus Raphidocystis. (XLSX)

## Abbreviations:

DIC: differential interference contrast
PhC: phase contrast
SEM: scanning electron microscopy
TEM: transmission electron microscopy
