# Supplementary figures and images for: Morphology and phylogeny of the centrohelid heliozoans Raphidocystidae and their ability to consume cyanobacteria
Source: PLoS One. 2025 May 9;20(5):e0322585. doi: 10.1371/journal.pone.0322585 (PMC12063867; doi:10.1371/journal.pone.0322585)

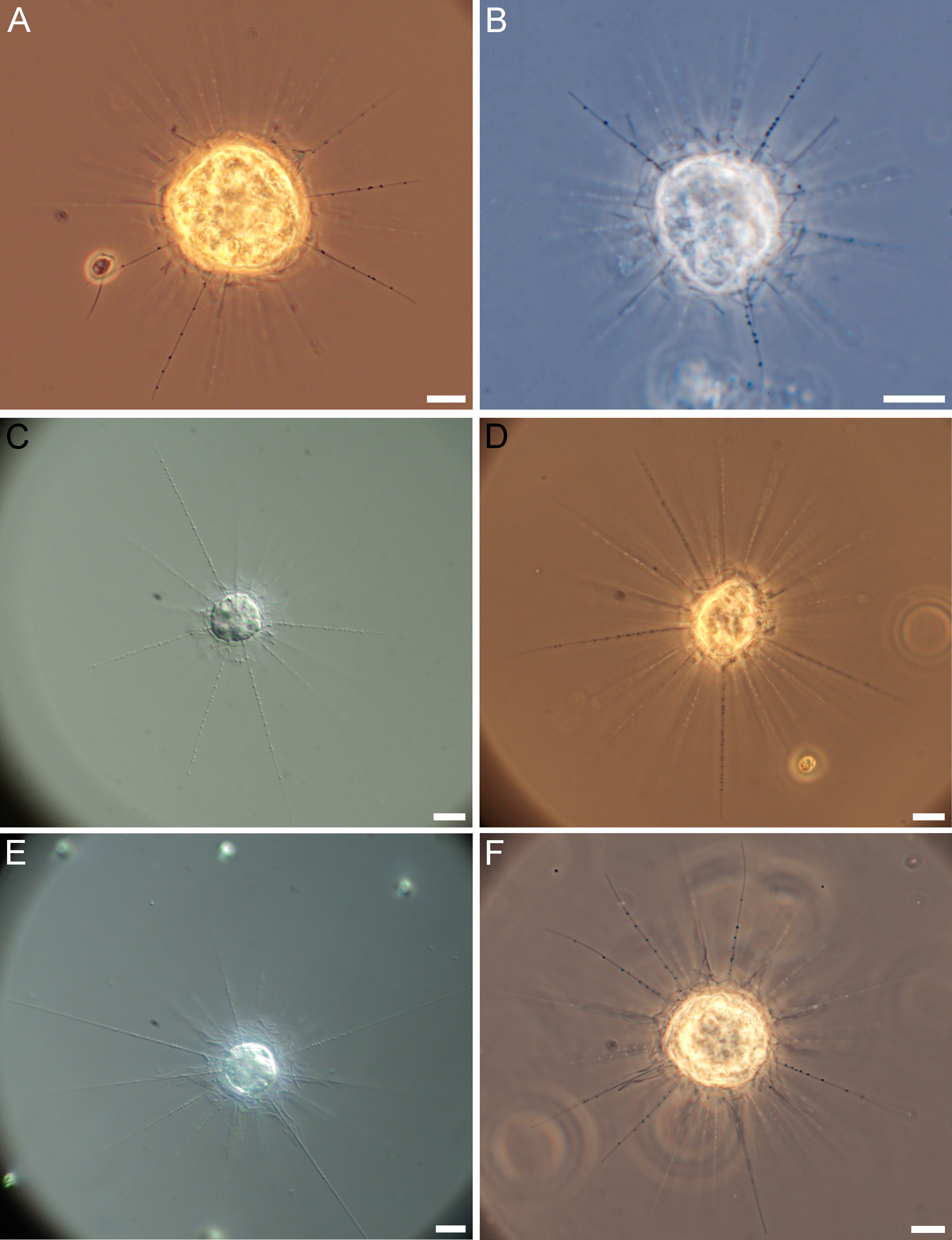

Supplement: S1 Fig — Raphidocystis tubifera (A–B), R. marginata (C–D), and R. symmetrica (E–F). A, B, D, F – PhC; C, E – DIC. Scale bars: 10 μm. (TIF) [file pone.0322585.s001.tif]
